# Progress toward improving outcomes in patients with cholangiocarcinoma

**DOI:** 10.1007/s11938-021-00333-2

**Published:** 2021-01-30

**Authors:** Hiroko Kawasaki, Yuko Akazawa, Nataliya Razumilava

**Affiliations:** 1 Department of Gastroenterology and Hepatology, Graduate School of Biomedical Sciences, Nagasaki University, Nagasaki, Japan;; 2 Department of Internal Medicine, University of Michigan, Ann Arbor, MI, USA

**Keywords:** personalized medicine, biliary cancer, immunotherapy, liver transplantation, FGFR2, IDH1/2

## Abstract

**Purpose of review:**

To provide an update on latest advances in treatment of cholangiocarcinoma.

**Recent findings:**

Incidence of cholangiocarcinoma has been increasing over the past decade. A better understanding of the genetic landscape of cholangiocarcinoma and its risk factors resulted in earlier diagnosis and treatment option expansion to targeted therapy with FGFR inhibitors, and liver transplantation for early perihilar cholangiocarcinoma and early intrahepatic cholangiocarcinoma. IDH1/2 inhibition for intrahepatic cholangiocarcinoma is an emerging targeted therapy approach. Data supports benefits of adjuvant therapy for a subset of patients undergoing surgical resection. Approaches combining different treatment modalities such as chemotherapy, surgery, radiation therapy appear promising.

**Summary:**

Earlier diagnosis and genetic characterization provided additional treatment options for patients with previously incurable cholangiocarcinoma. A precision medicine approach with a focus on actionable genetic alterations and combination of treatment modalities are actively being explored and will further improve outcomes in our patients with cholangiocarcinoma.

## Introduction

Cholangiocarcinomas (CCAs) are a group of rare tumors expressing biliary cell markers and associated with poor prognosis with a 5-year mortality rate of 95% [1, 2·]. The incidence of this second most common hepatobiliary cancer is increasing [3]. Based on anatomic location and biology, which determine prognosis and treatment strategy, CCAs are categorized as intrahepatic CCA (iCCA), perihilar CCA (pCCA) and distal CCA (dCCA) [4, 5]. Misclassification of pCCA as intrahepatic CCA or extrahepatic was commonly observed in the past. The American Cancer Society expects that 11,980 new patients will be diagnosed with biliary tract cancers (BTCs), including CCA and gallbladder cancer, and there will be 4,090 BTC-related deaths in the United States in 2020 [6·]. Surgical resection is a cornerstone of curative treatment for CCA, but this option can be offered only to a minority of patients. Systemic therapy with gemcitabine with platinum-based agents, which is offered to patients with advanced CCA, provides very modest improvement in patient survival. Lately, the treatment repertoire for CCA has expanded to molecular therapy targeting *FGRF2*, liver transplantation, use of adjuvant therapy, liver-directed therapies, and immune-therapies. This review will provide a background on CCA classification, epidemiology, and genetic aberrations, and an overview of contemporary therapies for CCA, which are established or under investigation.

### Classification and epidemiology

iCCA is localized to intrahepatic bile ducts and is proximal to the second-order intrahepatic branches, representing <10% of all CCA cases [7–9··]. Morphologically, iCCA can be mass-forming, periductal-infiltrating, intraductal, superficial spreading, and of undefined subtypes [5, 10]. The incidence of iCCA is rising [4, 11–15], thus, in the United States iCCA incidence increased by 5.9% between 2003 and 2009 [9··, 16]. pCCA arises between the second-order biliary ducts and the cystic duct origin, accounting for approximately half of CCAs [8]. pCCA is subclassified on mass-forming and intraductal types. The latter can be further divided into nodular or periductal infiltrating tumors with associated mass [2·, 5]. dCCA is formed in the region between the cystic duct origin and the papilla of Vater, accounting for almost 40 % of CCA cases [8]. Some studies report a stable or declining incidence of “extrahepatic CCAs,” which traditionally, but improperly, combine pCCA and dCCA.

The majority of CCAs are formed de-novo, while the risk factors for CCA include congenital choledochal cysts and stones, liver fluke, cirrhosis, and hepatitis B and C [2·]. Well-known risk factors of pCCA are primary sclerosing cholangitis (PSC), choledocholithiasis, and parasitic liver disease, such as liver flukes or liver schistosomiasis [2·, 17].

### CCA presentation and diagnosis

**Figure 1.**
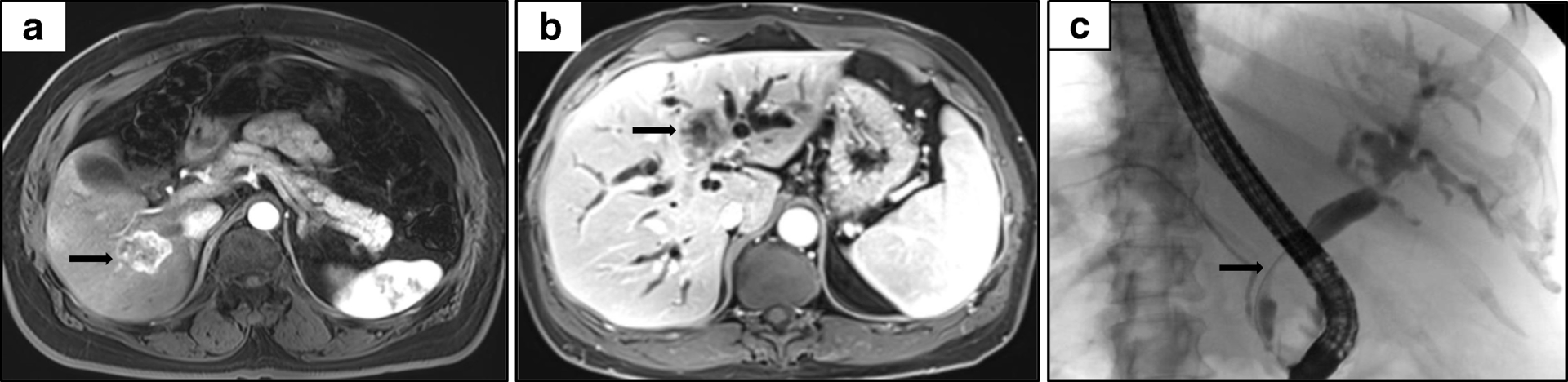
Imaging studies of the patients with intrahepatic (A), perihilar (B) and distal (C) cholangiocarcinoma. MRI demonstrates a mass (arrow) in the segment 5/6 of the liver that encases branches of the right portal vein and right hepatic artery (A). MRI shows prominent bilateral hepatic duct dilatation associated with a mass (arrow) anterior to the porta hepatis. The mass narrows the left portal vein (B). ERCP demonstrates left liver lobe biliary dilatation upstream of a common bile duct stricture (C) secondary to endoscopic biopsy-proven distal cholangiocarcinoma.

Patients with iCCA are often asymptomatic and present with a liver mass found incidentally or during liver cancer surveillance in patients at risk. Thus, ultrasound, cross-sectional abdominal computed tomography (CT), or magnetic resonance imaging (MRI) studies can reveal an intrahepatic lesion with features atypical for hepatocellular carcinoma (HCC) in patients with cirrhosis [5]. The asymptomatic nature of iCCA and its occurrence de-novo explains common diagnosis in late stages. On dynamic contrast-enhanced imaging studies, iCCA can appear as a mass with a ring enhancement during the arterial phase and increased progressive centripetal contrast enhancement during the venous phase (Figure 1A) [9··]. The presence of a mosaic pattern of early enhanced and non-enhanced areas on contrast-enhanced imaging studies should trigger a workup to exclude combined HCC-CCA, which has biological behavior distinct from CCAs behavior [18, 19].

Fluorine-18-fluorodeoxyglucose positron emission tomography (^18^F-FDG-PET) and PET/CT has high sensitivity and specificity (95% and 83%, respectively) for iCCA diagnosis [20]. Endoscopic ultrasound (EUS) is an effective tool for the detection of regional lymph nodes with sensitivity of 85%, which is only at 50% for cross-sectional studies (*P*=0.048) [21]. A biopsy is required for iCCA diagnosis and provides material for genetic analysis, considering that up to 50% of iCCA have targetable genetic alterations.

pCCA patients can present with cachexia, weight loss, and a mass on a cross-sectional study in patients at risk. Jaundice indicating biliary obstruction is a common presentation for both pCCA and dCCA [5]. In such cases, MRI with magnetic resonance cholangiopancreatography (MRCP) should be obtained and can demonstrate the biliary stricture with or without associated mass and upstream biliary obstruction (Figure 1B,C) [22]. A recent study has shown that surveillance with MRI with MRCP is associated with earlier diagnosis and improved outcomes in patients with PSC [23]. It is also important to differentiate dCCA from pancreatic cancer with a pancreatic head mass [5].

Endoscopic retrograde and percutaneous transhepatic cholangiography (ERC and PTC, respectively) can aid the pCCA and dCCA diagnosis, but cholangiogram-guided brushings still have a high false-negative rate, which sensitivity for pCCA is only 20–40% [5]. Notably, trans-peritoneal sampling of the mass in pCCA, including with fine-needle aspiration, should be avoided if liver transplantation is contemplated due to the high risk of track seeding [24]. The diagnosis of pCCA is challenging and requires the positive cytology brushings; positive transcatheter biopsy; polysomy on cytological analysis with fluorescence *in situ* hybridization; carbohydrate antigen 19–9 > 100 U/ml; or a hilar mass on cross-sectional imaging at the site of the malignant appearing stricture [7]. EUS aids in pCCA and dCCA staging with lymph nodes evaluation in pCCA and dCCA [5].

### Genetic landscape of CCAs

Progress in understanding of the genetic landscape of CCA has opened opportunities for personalized medicine. 40–50% of CCAs are thought to have “actionable” genetic alterations [25··, 26]. Notably, CCA subtypes have a distinct genetic landscape. In iCCA, commonly mutated genes, based on MSK-IMPACT platform analysis, include isocitrate dehydrogenase 1 (*IDH1*; 30%), AT-rich interaction domain 1A (*ARID1A*; 23%), BRCA1 associated protein 1 (BAP1, 20%), *TP53* (20%), as well as fibroblast growth factor receptor 2 (*FGFR2*) gene fusions (14%) [27]. Targetable genetic alterations in *IDH1/2*, *FGFR*, and *BAP1* are specific and diagnostic for iCCA [28]. A recent study has shown the genetic landscape of CCAs also depends on underlying liver disease and pathogenesis. Thus, fluke-positive CCAs are reported to bear frequent *ERBB2* amplifications and *TP53* mutations compared to non-fluke-associated CCAs [28].

Information regarding the genetic landscape of pCCA and dCCA has been limited and often combines pCCA and dCCA together. *KRAS* (36.7%), *TP53* (34.7%), *ARID1A* (14.0%) and *SMAD4* (10.7%) were shown to be the most prevalent genetic alterations in both pCCA and dCCA [29]. *KRAS* and *TP53* mutations have higher prevalence in pCCA and dCCA as compared with iCCA [29]. Currently, actionable genetic mutations in extrahepatic CCAs are limited to 3% in *BRCA1/2* (3%) and the epidermal growth factor receptor (*EGFR*; 1%) [29].

## Treatment

### Surgical resection

A surgical resection with negative margins (R0) is the best treatment option for patients with CCA (Table 1) [30, 31]. However, the majority of patients with iCCA have advanced disease and only 35% of patients have resectable tumors [32]. The 5-year overall survival (OS) rate of patients with multifocal iCCA has been reported at 9.9%, which is significantly lower compared to patients with solitary tumors (49.4 %) and tumors with satellites in the same liver segment (34.2%; *P*=0.021) [33].

To prevent surgery-related morbidity and mortality, it is pivotal to take into consideration a future liver remnant volume. At least 25% of the pre-operative liver volume in the normal liver or 30–40% of pre-operative liver volume in patients with chronic liver diseases should remain after resection [34]. Portal vein embolization (PVE) is frequently employed to enhance a potential remnant volume, and is achieved by embolization of the portal vein on the side of the liver that is resected [35]. Associating liver partition and portal vein ligation for staged hepatectomy (ALPPS) is a new technique enable remnant hypertrophy [36–38]. The first step of an ALPPS procedure involves portal vein ligation with parenchymal liver transection at a planned resection site to induce hypertrophy of a future liver remnant. One to two weeks later, the actual liver resection is performed and tumor is removed. While the initial experience with ALPPS was associated with high morbidity and mortality, procedure refinement has led to its wider acceptance to treat locally advanced iCCA [39].

During iCCA resection, portal lymph node dissection and retrieval of more than six lymph nodes is recommended for accurate staging by the latest edition of the National Comprehensive Cancer Network (NCCN) and American Joint Committee on Cancer (AJCC) [40]. Accumulating data indicates that laparoscopic hepatectomy can be a safe and practical option for iCCA, which has a faster recovery and is comparable with the R0 rates, OS and disease-free survival similar to open surgery [41]. The accuracy of laparoscopic regional lymphadenectomy is less defined.

Hemi-hepatectomy is usually required for surgical treatment of pCCA [34] and is technically difficult due to tumor location. Requirements for biliary drainage before pCCA surgery to optimize a remnant function and patient recovery has not been standardized [34]. When the expected liver remnant is <40%, most centers would employ biliary drainage prior to PVE to relieve biliary obstruction and facilitate liver regeneration [34]. Drainage of atrophic liver segments is not required and rather can increase the risk of infection.

PVE before major liver resection for pCCA has been reported to reduce liver failure, postoperative complications, and mortality [42]. The data suggests that outcomes in patients with pCCA undergoing liver resection with ALPPS are suboptimal with 90-day mortality and median OS at 48% and 6 months, respectively, in those with ALPPS, compared to 24% and 27 months, respectively, in those without ALPPS [43]. A modified technique that minimizes invasiveness of ALPPS had been proposed and requires further evaluation [44, 45].

The Whipple procedure, which includes pancreaticoduodenectomy with removal of the head of the pancreas, is a principal surgical approach in patients with dCCA [5]. When the R0 status is achieved, the 5-year recurrence rate remains high at >50% [46]. Main predictors of recurrence and the OS are perineural and/or pancreatic invasion and positive lymph nodes [46]. The American Society of Anesthesiologist physical status classification (*P*<0.001), tumor grade (*P*=0.009), and margin status (*P*=0.042) are additional factors influencing the OS [47]. Emerging data indicate that multimodal treatment can improve patient survival. Thus, one study from a single institution reported the median survival in the surgery alone group to be 21.9 months, whereas the surgery/chemotherapy group survived 34.3 months, and the surgery/chemoradiation group survived 69.1 months [48].

### Liver transplantation

Orthotopic liver transplantation (OLT) with neoadjuvant chemoradiation is curative treatment for a highly selected subset of patients with unresectable pCCA tumors that are ≤3 cm in diameter, and without metastases [7]. OLT for early pCCA is associated with the median survival rate of 53 months and the 5-year survival rate of 55% [49].Neoadjuvant chemoradiation involves external-beam radiation with continuous infusion of 5-flurouracil (5-FU) followed by brachytherapy. Patients receive oral capecitabine until the time of transplantation preceded by an exploratory laparotomy to exclude metastases [7]. Patients with PSC-associated pCCA derive most benefits after OLT for pCCA with the longest survival (adjusted R^2^=0.82, *P*=0.007) [49]. An ongoing prospective study compares liver resection versus OLT with neoadjuvant chemoradiation for pCCA [TRANSPHIL (NCT02232932)] [50].

Living donor liver transplantation can be an additional option expanding the donor organ pool for OLT at centers with available expertise. However, in patients with PSC versus other indications, the rate of late vascular complications involving hepatic artery (18.9% versus 4.1%; *P*<0.001) and portal vein (37.8% versus 8.7%; *P*<0.001) are more frequent. Interestingly, these complications do not significantly affect long-term survival after OLT for PSC-pCCA with the overall 1-, 5-, and 10-year survival at 84.9%, 66.5%, and 55.6%, respectively [51].

In contrast to pCCA, OLT is not a standard of practice for iCCA due to the still high post-operative recurrence rate and low OS [52, 53]. However, recent studies suggest its utility for early stage, small solitary, and/or well-differentiated tumors [54··–56]. Thus, the 5-year survival of cirrhotic patients after OLT who had single ≤2 cm iCCA is 65–73% versus 45% in ≥2 cm or multifocal iCCA [55, 57]. The tumor size, tumor volume, microscopic invasion, and poor degree of differentiation are predictors of the tumor recurrence after OLT.

### Liver-directed therapies

Radiofrequency ablation (RFA), microwave ablation (MWA), cryoablation, transarterial chemoembolization (TACE), transarterial radioembolization (TARE), hepatic arterial infusion (HAI) and stereotactic body radiation therapy (SBRT) can be offered to some patients with localized and unresectable iCCA [4, 58, 59]. However, due to rich desmoplastic stroma and less prominent vascularization in CCA, catheter-based therapies might have less utility than in HCC.

The indications for RFA in iCCA have not been clearly determined but are deemed to be most effective for ≤3 cm tumors [60]. In HCC and hepatic metastases, MWA has advantages over RFA for >3 cm lesions or when the tumor is in a close proximity to the large blood vessels [61]. MWA for primary iCCA and recurrence after resection iCCA was associated with a 5-year OS and 3-year recurrence-free survival (RFS) rate of 23.7% versus 33.1% and 21.8% versus 30.6%, respectively [62]. The meta-analysis of studies on iCCA ablation reported the median OS at 8.7–52.4 months and the pooled 1-year and 5-year survival rates at 76% and 16%, respectively [3].

In one study, endoscopic RFA combined with biliary stenting has been shown to be more beneficial than stenting alone for unresectable pCCA with the mean OS at 13.2 ± 0.6 months for RFA/stenting group versus 8.3 ± 0.5 months for the stent-only group (*P*<0.001). The stent patency was also prolonged with combination therapy (6.8 versus 3.4 months in a RFA/stenting and stent-alone group, respectively, *P*=0.02) without an increase in adverse events [63].

Conventional TACE (cTACE) involves administration of the lipiodol-chemotherapeutic agent suspension and gelatin sponge particles [64]. Despite lower iCCA vascularization as compared to HCC, cTACE versus symptomatic management alone was shown to be beneficial in unresectable iCCA with the median survival at 12.2 versus 3.3 months, respectively (*P*<0.001) [65]. TACE with irinotecan-eluting beads (iDEB-TACE) demonstrated better outcomes than cTACE with the progression-free survival (PFS) and OS at 3.9 months and 11.7 months versus 1.8 months and 5.7 months, respectively [66]. A presence of the bilio-enteric anastomosis, biliary stent, hypoalbuminemia, portal vein invasion, and history of sphincterotomy are main risk factors for liver abscess after TACE [59, 67]. Ytterium-90 (^90^Y) radioembolization for iCCA has shown similar outcomes with systemic chemotherapy and TACE with the OS at 15.5 months [68] [69]. TARE for recurrent after resection iCCA was associated with significantly shorter survival compared to TARE for primary iCCA (3.9 versus 12.8 months, *P*=0.002) with no cases of radiation-induced liver injury in 37 treated patients [70]. Overall, TARE appears to be a safe option for local tumor control in patients with primary iCCA.

During HAI, the chemotherapeutic agent is directly delivered into the hepatic artery via a surgically implanted pump with a percutaneously placed catheter [71]. This approach had been used in patients with colorectal liver metastasis [58] and was recently adopted for patients with unresectable iCCA. The key contraindications to HAI are portal hypertension or portal inflow obstruction [58]. A single-arm phase 2 clinical trial of fluoxuridine (FUDR) HAI in combination with systemic gemcitabine and oxaliplatin showed that among 38 treated iCCA patients, 84% achieved disease control at 6 months with the median PFS and 1-year survival rate of 11.8 months and 89.5%, respectively, suggesting acceptable tolerance and efficacy [72]. A pilot study examining HAI of cisplatin plus oral TS-1 (a novel oral fluoropyrimidine preparation) in patients with unresectable iCCA reported a significant extension of the OS as compared with “other” treatments, including radiation, TACE, and systemic chemotherapy, group (10 versus 4 months, respectively) [73]. A prospective phase 2 trial of HAI with oxaliplatin and 5-FU for patients with advanced unresectable pCCA has shown a high tumor control rate (89.2%) and median PFS and OS (12.2 and 20.5 months, respectively) [74].

SBRT has shown effectiveness for iCCA and also extrahepatic CCA with the pooled 1-year and 2-year OS, and 1-year local control rate of 58.3%, 35.3%, and 83.4%, respectively [75]. The effectiveness of combinations of chemotherapy, chemoradiation, and SBRT still needs to be investigated. A large randomized-controlled trial of photodynamic therapy (PDT) for BTCs with obstructive jaundice showed inferior outcomes of PDT to stenting alone with the median OS at 6.2 versus 9.8 months (HR 1.56, 95% CI 1.00–2.43, *P*=0.048) and the median progression-free survival at 3.4 versus 4.3 months (HR 1.43, 95% CI 0.93–2.18, *P*=0.10), respectively [76]. Thus, PDT is not currently recommended for CCA.

## Systemic therapy

Chemotherapy is indicated for patients with advanced CCA, who are not candidates for surgical resection, liver transplantation, or liver-directed therapies. The current first-line standard of practice for CCAs is a combination of gemcitabine and cisplatin. Its approval in 2010 was based on the Advanced Biliary Cancer (ABC)-02 study results showing 3 months prolongation of the median OS and PFS compared to gemcitabine alone [77]. A recent study comparing an active symptom control with and without second-line therapy with oxaliplatin/L-folinic acid/5-FU (mFOLFOX) after progression to first-line cisplatin and gemcitabine (ABC-06 study) showed a modest but increased median OS (6.2 versus 5.3 months, respectively) [78, 79]. There also were more patients still alive after one year in the chemotherapy group.

A randomized, controlled, multicenter phase III study on capecitabin compared with observation in resected BTC (BILCAP) demonstrated a longer median OS in the per-protocol analysis (53 versus 36 months, respectively, adjusted HR 0.75, 95% CI 0.58–0.97; P=0.028) and longer median RFS (25.9 versus 17.4 months, respectively, 95% CI 19.8–46.3 and 12.0–23.7, respectively) [80, 81]. The prospective randomized phase III study on adjuvant strategies using gemcitabine/cisplatin in BTC patients following a curative intent surgery (ACTICCA-1, NCT02170090) [6·, 68] and on adjuvant S-1 therapy versus observation alone in resected BTC (JCOG1202, ASCOT) are currently underway [82].

Clinical trials on first-line treatments in advanced settings also are ongoing. One is a randomized controlled multicenter phase II/III study on modified FOLFIRINOX versus cisplatin and gemcitabine (CisGem) as first-line chemotherapy for locally advanced unresectable or metastatic BTC [(AMEBICA)-PRODIGE38] [83]. Another is an interventional, prospective, randomized, controlled, open label, two-sided phase II study comparing nanoliposomal-irinotecan (nal-IRI) with 5-FU/leucovorin and gemcitabine plus cisplatin as a first-line chemotherapy in advanced BTC (NIFE, AIO-YMO HEP-0315) [84].

## Molecular therapy

Current CCA treatment approaches are mainly “location-based.” However, progress with molecular and immunotherapy will likely change the selection of CCA treatment to “genetic and molecular alteration-based,” regardless of the tumor location. The emerging molecular targeted therapies are presented below.

### FGFR inhibitors

Inhibition of Fibroblast Growth Factors (FGFs) and their cognitive receptors is of special interest. An efficacy and safety study of pemigatinib, a protein kinase inhibitor, in subjects with advanced/metastatic or surgically unresectable CCA who failed in previous therapy was evaluated in the FIGHT-202 clinical trial [85··]. In this trial, pemigatinib was associated with a complete response in 2.8%, partial response in 32.7%, stable disease in 46.7%, and progressive disease in 14.9% of the patients who had FGFR2 fusions/rearrangements. The PFS with pemigatinib was 6.9 months. Based on the FIGHT-202 trial, the United States Food and Drug Administration (FDA) granted an accelerated approval for previously treated unresectable locally advanced CCA with FGFR2 fusion or other rearrangements [86].

A phase III, open-label, randomized study is now being conducted to evaluate first-line pemigatinib versus gemcitabine-cisplatin in a FGFR2 rearrangement-bearing unresectable or metastatic CCA (FIGHT-302; NCT03656536). Another trial comparing irreversible FGFR1–4 inhibitor, futibabinib, with gemcitabine-cisplatin as a first-line therapy for patients with advanced CCA bearing FGFR2 gene rearrangements (FOENIX-CCA3) also is in phase III [87].

In FGFR2 fusion-positive iCCA, use of the multi-kinase inhibitor derazantinib (ARQ 087) resulted in an estimated median PFS of 5.7 months (95% CI: 4.04–9.2 months) with tolerable side effects during an open-label phase I/II study [88]. In a phase II trial of BGJ398, a selective pan-FGFR kinase inhibitor, the treatment was reported to exert a similar estimated median PFS of 5.8 months (95% CI, 4.3 to 7.6 months) [89].

### Targeting metabolic regulators in CCA

Genetic alterations in a gene encoding metabolic regulator IDH1/2 is common in iCCAs [90··] [29] and is targetable. In the phase III ClarIDHy trial, ivosidenib, small molecule inhibitor of mutant IDH1, improved PFS over a placebo (2.7 versus 1.4 months, respectively) in IDH1-mutant chemotherapy-refractory CCA, but no improvement in the median OS was observed [91··]. The PFS rates with ivosidenib at 6- and 12-months were 32% (95% CI 23–42) and 22% (95% CI 13–32), respectively. While no patients in the placebo group were progression-free [91··].

Other molecular targets include EFGR (HER2; cetuximab, lapatinib, erlotinib) and VEGFR (bevacizumab). MEK1/2 showed limited efficacy in phase I and II studies [90··].

### Targeting the DNA damage

BAP1 regulates cellular differentiation, cell death, and DNA damage response (DDR), and is a tumor suppressor [92]. BAP1 is mutated, leading to a loss of functional protein, in ~25% of iCCAs [93, 94]. A phase II trial of niraparib, the inhibitor poly(adenosine diphosphate-ribose) polymerase (PARP), which is important for BAP1-mutant cancers survival, is in progress for patients with CCA (NCT03207347) [95]. IDH-mutant CCAs also display increased DNA damage and might potentially benefit from DDR modulation. Thus, PARP inhibitors AXD6738 (NCT03878095) and olaparib (NCT0321227) are now in phase II clinical trials for IDH-mutated CCA.

### Targeting cancer immunity

Cancers develop immune escape mechanisms by creating immune checkpoint proteins, including Programmed-death-1(PD-1) or its ligand, Programmed-death Ligand 1 (PD-L1), inserting an immunosuppressive effect. Monoclonal antibodies targeting immune checkpoint proteins has emerged as an effective systemic therapy option in solid tumors [96, 97].

Although the majority of iCCAs and pCCAs are negative for PD-1 or PDL-1, these protein expressions in CCA have been associated with poor OS in CCA patients [97]. The use of pembrolizumab, a PD-1 inhibitor, in KEYNOTE-158 (NCT02628067; phase II) and KEYNOTE-028 (NCT02054806; phase Ⅰ b) trials in patients with advanced BTC with one or more lines of previous therapy has returned an objective response rate (ORR) of 5.8% and 13.0%, respectively, with a manageable safety profile [98·]. However, information on a specific tumor location has not been available in these studies.

A combination of pembrolizumab with ramucirumab (VEGFR2 inhibitor) for BTCs showed very limited activity with ORR 4% [99]. While in the single-center observational study of 14 patients with advanced iCCA previously treated with more than two lines of therapy, a combination of lenvatinib, a multikinase inhibitor targeting VEGFR 1–3, with PD-1 inhibitors pembrolizumab and nivolumab ORR, a partial response rate was observed in 21% of patients [100]. The high tumor mutational burden was strongly associated with a better response to combination therapy [100].

### Supportive care

Therapy without curative intent is considered palliative, and includes loco-regional and systemic therapies, palliative resection, and biliary drainage with endoscopic, preferably, or percutaneous stenting. Biliary stenting can improve liver function and performance status for chemotherapy or to alleviate symptoms of pruritus, malabsorption, and to prevent infection. In patients with palliative stenting and life-expectancy beyond 4–6 months, metal stents are preferred over plastic stents as they provide better durability, decrease frequency of invasive procedures, and are more cost-effective [7]. Active symptom control might be more beneficial than best supportive care as aggressive surveillance for biliary obstruction, pain, and infection addresses them earlier and might improve quality of life.

## Conclusions

Therapeutic options for hard-to-treat CCA are expanding. A better understanding of risk factors for CCA, like cirrhosis and viral hepatitis, increases awareness of CCA in patients undergoing cancer surveillance. This eventually should lead to diagnosis in earlier actionable stages. FGFR2-targeted therapy has been recently FDA approved for patients with *FGRF2* genetic aberrations who progressed on first line therapy. Techniques enhancing the liver remnant volume make surgical resection feasible for patients with iCCA and pCCA, who previously were not surgical candidates. While adjuvant chemotherapy might further extend the OS in patients undergoing liver resection. Liver transplantation with neoadjuvant chemoradiation provides cure for a subset of patients with pCCA and emerges as a new treatment option for patients with early iCCA. Living donor OLT expands the donor organ pool. The active symptom control with frequent surveillance and treatment of biliary-related complications, including stenting and antibiotics, should be considered for patients who are not candidates for other therapies.

Overall, as clinicians, we have many more options now to improve the outcomes of our patients with CCA. Recent efforts in decoding genetic mutations in CCAs will hopefully allow a personalized medicine approach for each patient and their specific cancer in the future.

## Figures and Tables

**Table 1.** Treatment options for subtypes of cholangiocarcinoma (CCA). Fibroblast growth factor receptor 2 (FGFR2), oxaliplatin/L-folinic acid/5-fluorouracil (mFOLFOX).

CCA subtype	Therapy
**Intrahepatic CCA**	Surgical resection Systemic therapy with gemcitabine and cisplatin or oxaliplatin for advanced cancer Pemigatinib for previously treated advanced CCA with genetic derangements in *FGFR2* Emerging therapies: Liver transplantation for early unifocal ≤2 cm tumor Adjuvant therapy with capecitabine mFOLFOX as a second line therapy Liver-directed therapies
**Perihilar CCA**	Surgical resection Liver transplantation with neoadjuvant chemoradiation Systemic therapy with gemcitabine and cisplatin or oxaliplatin along with biliary stenting for unresectable cancer
**Distal CCA:**	Surgical resection Systemic therapy with gemcitabine and cisplatin or oxaliplatin along with biliary stenting for unresectable cancer

## List of Abbreviations:

CCA: cholangiocarcinomas
iCCA: intrahepatic CCA
pCCA: perihilar CCA
dCCA: distal CCA
BTC: biliary tract cancers
PSC: primary sclerosing cholangitis
HCC: hepatocellular carcinoma
CT: computed tomography
MRI: magnetic resonance imaging
EUS: endoscopic ultrasound
ERC: endoscopic retrograde cholangiography
PTC: percutaneous transhepatic cholangiography
CA 19–9: carbohydrate antigen 19–9
IDH1: isocitrate dehydrogenase 1
ARID1A: AT-rich interaction domain 1A
FGFR2: fibroblast growth factor receptor 2
EGFR: epidermal growth factor receptor
OS: overall survival
PVE: portal vein embolization
cHCC-CCA: combined hepatocellular-cholangiocarcinoma
ALPPS: associating liver partition and portal vein ligation for staged hepatectomy
NCCN: National Comprehensive Cancer Network
AJCC: American Joint Committee on Cancer
OLT: orthotopic liver transplantation
RFA: radiofrequency ablation
MWA: microwave ablation
TACE: transarterial chemoembolization
TARE: transarterial radioembolization
HAI: hepatic arterial infusion
SBRT: stereotactic body radiation therapy
RFS: recurrence-free survival
cTACE: conventional TACE
PDT: photodynamic therapy
PFS: progression free survival
BAP1: BRCA1-Associated Protein 1
DDR: DNA damage response
PARP: the inhibitor poly (adenosine diphosphate-ribose) polymerase
PD-1: programmed-death-1
PD-L1: programmed-death ligand 1
ORR: objective response rate
